# Association of Serum Free Fatty Acids with Hypertension and Insulin Resistance among Rural Uyghur Adults in Far Western China

**DOI:** 10.3390/ijerph120606582

**Published:** 2015-06-09

**Authors:** Shu-Xia Guo, Yi-Zhong Yan, La-Ti Mu, Qiang Niu, Jia He, Jia-Ming Liu, Shu-Gang Li, Jing-Yu Zhang, Heng Guo, Dong-Sheng Rui

**Affiliations:** 1 Department of Public Health, Shihezi University School of Medicine, Shihezi, Xinjiang 832000, China; E-Mails: yyz19880215@sina.com (Y.-Z.Y.); 464174936@qq.com (L.-T.M.); niuqiang1214@163.com (Q.N.); hejia123.shihezi@163.com (J.H.); 15542815@qq.com (J.-M.L.); lishugang@ymail.com (S.-G.L.); yfyxxzjy@126.com (J.-Y.Z.); guoheng@shzu.cn.com (H.G.); ruidongsheng@gmail.com (D.-S.R.); 2 Department of Pathology, Key Laboratory of Xinjiang Endemic and Ethnic Diseases (Ministry of Education), Shihezi University School of Medicine, Shihezi, Xinjiang 832000, China

**Keywords:** Uyghur, hypertension, IR, FFA

## Abstract

*Objective*: We aimed to investigate whether free fatty acid (FFA) levels in hypertensive patients result from increased blood pressure or the accompanying condition of insulin resistance (IR). Furthermore, we aimed to study the role of IR in the onset and development of hypertension and then provide a basis for the prevention and treatment of hypertension and metabolic syndrome (MS). *Design and Methods*: The study included 68 essential hypertensive patients without IR, 87 normotensive subjects with IR, 82 essential hypertensive patients with IR and 74 normotensive subjects without IR. Analysis of covariance was used to compare FFA concentrations among the four groups to determine the association between FFA concentrations and hypertension and IR. *Results*: A significant difference in FFA concentrations among the four groups was found using a one-factor analysis of variance (*p* < 0.001). A significant difference was also found among the adjusted means of the four groups (essential hypertensive patients with IR *vs.* normotensive subjects without IR: 0.703 mg/L *vs.* 0.516 mg/L, *p* < 0.001; essential hypertensive patients with IR *vs.* normotensive subjects with IR: 0.703 mg/L *vs.* 0.525 mg/L, *p* < 0.001; essential hypertensive patients with IR *vs.* essential hypertensive patients without IR: 0.703 mg/L *vs.* 0.579 mg/L, *p =* 0.002; normotensive subjects with IR *vs.* normotensive subjects without IR: 0.525 mg/L *vs.* 0.516 mg/L, *p =* 0.007; essential hypertensive patients without IR *vs.* normotensive subjects without IR: 0.579 mg/L *vs.* 0.516 mg/L, *p <* 0.001). However, no significant interaction was detected between IR and hypertension regarding the FFA concentration. *Conclusions*: FFA is an independent factor for IR and hypertension among Uyghur adults in a rural area of Xinjiang.

## 1. Introduction

Free fatty acids (FFAs) are endocrinological mediators that play an important role in the regulation of glucose and lipid metabolism and insulin sensitivity [1]. Elevated levels of lipids such as FFAs and triglycerides (TCs) are a key reason for the incidence of insulin resistance (IR) [2]. IR contributes to the prevalence of obesity, hypertension and dyslipidemia and can ultimately induce metabolic syndrome (MS) and other related diseases. Additionally, FFAs may be associated with hypertension, although it remains to be confirmed whether FFAs represent an independent or dependent factor for hypertension.

China is a multi-ethnic country. More than 10 distinct ethnic groups reside in the Xinjiang Uyghur Autonomous Region, with the Uyghur being one of the largest inhabitant minority groups, and most of these individuals reside in low-income rural communities [3]. Due to a poor economy, limited public health resources and inadequate transportation, few serious investigations have analyzed local public health needs in the region, including the underlying reasons for hypertension, IR and related diseases such as diabetes and cardiovascular diseases (CVDs). Our previous studies showed that increased FFA levels affected the incidence of hypertension and were an independent risk factor for hypertension among Kazakhs in far Western China [4]. However, due to differences in religion, culture, lifestyle, diet, and genetic background, it has yet to be determined whether this relationship is also to be found for the Uyghur population. In this study, we analyzed the relationship between FFAs, hypertension and IR among Uyghurs residing in far Western China to determine whether FFAs are an independent factor for hypertension and IR and to gain a better understanding of the incidence of hypertension and IR in Xinjiang.

## 2. Materials and Methods

### 2.1. Ethics Statement

The Institutional Ethics Review Board (IERB) at the First Affiliated Hospital of Shihezi University School of Medicine approved the study (IERB No. SHZ2010LL01). Standard university hospital guidelines, including informed consent, voluntary participation, confidentiality, and anonymity, were followed. All participants provided their written informed consent before the study began.

### 2.2. Settings and Participants

This study was conducted from 2009 to 2012 among Uyghurs living in the Bazi Xiang River region of Jiashi and Kashi in Xinjiang. We investigated MS among Uyghur residents who were ≥18 years old. Based on the baseline investigation, we randomly selected a total of 311 hypertensive patients and normal people as the subjects for laboratory testing using a random number table generated in SPSS version 19.0. The subjects were divided according to the diagnostic criteria of hypertension and IR into four groups as follows: 68 essential hypertensive patients without IR, 87 normotensive subjects with IR, 82 essential hypertensive patients with IR and 74 normotensive subjects without IR.

### 2.3. Definition of MS and HOMA-IR

(1) Hypertension was defined as follows: systolic blood pressure (SBP) ≥ 140 mmHg and/or diastolic blood pressure (DBP) ≥ 90 mmHg [5].

(2) The homeostatic model assessment of insulin resistance (HOMA-IR) was defined as follows: fasting insulin (in micro-international units (µIU) per mL) × fasting glucose (in mM)/22.5 [6]. The Chinese Diabetes Society (CDS) considers that IR can be estimated by this formula in epidemiological or clinical studies, with the upper quartile of subjects being the split point [7].

### 2.4. Exclusion Criteria

Patients who: (1) had serious heart and liver dysfunction; (2) were currently using insulin and oral hypoglycemic, antihypertensive, or lipid-lowering drugs; (3) were pregnant; (4) or had cancer, tuberculosis and infection diseases were excluded.

### 2.5. Laboratory Tests

(1) Total cholesterol (TC), triglycerides (TGs), low-density lipoprotein cholesterol (LDL-C), high-density lipoprotein cholesterol (HDL-C), and fasting glucose were analyzed using a biochemical autoanalyzer (Olympus AU 2700, Olympus Diagnostics, Hamburg, Germany) in the clinical laboratory; (2) FFA levels were determined by a colorimetric assay using kits purchased from Randox Laboratories Ltd. (Shanghai, China). Insulin was measured by radioimmunoassays using kits purchased from Beijing Atomic-Tech Co., Ltd. (Beijing, China).

### 2.6. Statistical Analysis

All analyses were performed with the SPSS statistical package for Windows, Version 19.0. (SPSS Inc., Standford, CA, USA). Continuous and normally distributed variables were presented as the means ± standard deviations (M ± SD) and analyzed using analysis of variance and covariance; the constituent gender ratio was compared using a chi-square test. Differences with *p* < 0.05 were considered statistically significant.

## 3. Results

### 3.1. Balancing Tests for SBP, DBP and HOMA-IR among the Four Groups

To ensure comparability among the four groups, we tested SBP and DBP among groups with or without hypertension and HOMA-IR among groups with or without IR. The distributions of SBP, DBP and HOMA-IR were balanced between the corresponding groups (*p* > 0.05 for each comparison) (Table 1).

**Table 1:** Balancing tests for SBP, DBP and HOMA-IR among the four groups.

Index	With IR (*n* = 87)	With Hypertension (*n* = 68)	With Hypertension and IR (*n* = 82)	Normal (*n* = 74)	*p*
SBP	-	145.35 ± 13.24	146.90 ± 18.21	-	0.813
	111.06 ± 10.24	-	-	114.26 ± 9.82	0.069
DBP	-	92.37 ± 11.12	94.78 ± 12.51	-	0.996
	71.15 ± 8.87	-	-	72.91 ± 7.42	0.223
HOMA-IR	-	0.68 ± 0.28	-	0.69 ± 0.34	0.935
	4.02 ± 2.36	-	4.22 ± 2.34	-	0.786

Notes: IR = insulin resistance, SBP = Systolic blood pressure, DBP = Diastolic blood pressure, HOMA-IR = homeostatic model assessment of insulin resistance.

### 3.2. One-Factor Analysis of Variance

Age, BMI, TGs, TC, HDL-C, LDL-C and FFAs were compared among the four groups by one-factor analysis of variance, and the constituent gender ratio was assessed using a chi-square test. Age, BMI, TGs, TC, HDL-C, LDL-C and FFAs were different among the four groups (*p* < 0.05 for each comparison), but no significant difference was identified for gender (*p* > 0.05) (Table 2).

**Table 2:** Index comparisons among the four groups.

Index	With IR	With Hypertension	With Hypertension and IR	Normal	*χ^2^*/*F*	*p*
	(*n* = 87)	(*n* = 68)	(*n* = 82)	(*n* = 74)		
Gender (male/female)	46/41	38/30	48/34	36/38	1.651	0.648
Age (year)	39.30 ± 11.67	44.05 ± 10.91	45.43 ± 11.53	40.13 ± 11.67	4.883	0.003
BMI (kg/m^2^)	22.93 ± 3.50	24.58 ± 3.84	25.83 ± 3.79	22.06 ± 2.63	16.070	<0.001
TG (mmol/L)	1.41 ± 0.98	1.35 ± 0.86	1.70 ± 0.97	1.02 ± 0.57	6.402	<0.001
TC (mmol/L)	4.54 ± 1.04	4.75 ± 1.33	4.74 ± 0.96	4.22 ± 1.09	2.994	0.031
HDL-C (mmol/L)	1.19 ± 0.29	1.16 ± 0.30	1.08 ± 0.29	1.29 ± 0.26	5.796	0.001
LDL-C (mmol/L)	2.48 ± 0.84	2.67 ± 0.67	2.70 ± 0.62	2.32 ± 0.54	3.922	0.009
FFA (mg/L)	0.52 ± 0.15	0.58 ± 0.25	0.73 ± 0.22	0.39 ± 0.11	23.614	<0.001

Notes: IR = insulin resistance, BMI = body mass index, TG = triglyceride, TC = total cholesterol, HDL-C = high-density lipoprotein cholesterol, LDL-C =low-density lipoprotein cholesterol, FFA = free fatty acid.

### 3.3. Analysis of Covariance

**Figure 1:**
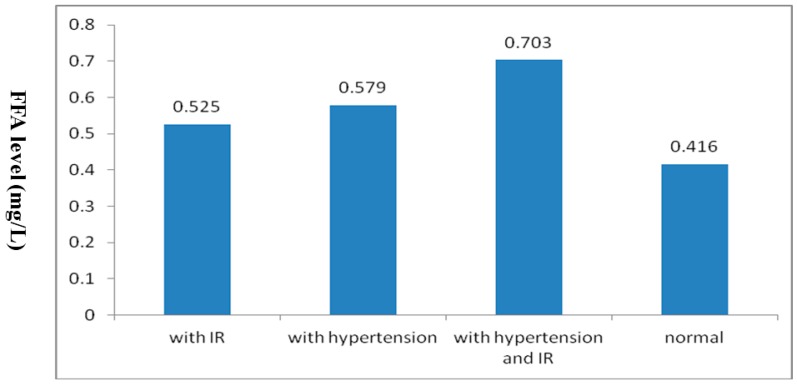
Comparisons of adjusted means among the four groups.

To exclude interference by confounding factors such as age, BMI, TGs, TC, HDL-C and LDL-C when comparing FFA levels among the four groups, we used analysis of covariance with age, BMI, TGs, TC, HDL-C and LDL-C as covariates, all of which were statistically significant by one-factor analysis of variance with the FFA level as the dependent variable. The adjusted means of FFA levels significantly differed among the four groups (*F* = 10.082, *p* < 0.001). Additionally, by multiple comparisons, any two groups were significantly different (*p* < 0.05 for each comparison), except for the group with IR compared with another group with hypertension. (Figure 1, Table 3).

**Table 3:** Multiple comparisons of adjusted mean FFA levels.

Groups	*p*	95% CI
With hypertension and IR/with IR	<0.001	(0.114, 0.243)
With hypertension and IR/with hypertension	0.002	(0.048, 0.201)
With hypertension and IR/normal	<0.001	(0.013, 0.162)
With IR/normal	0.007	(0.039, 0.277)
With hypertension/normal	<0.001	(0.102, 0.247)
With IR/with hypertension	0.170	(−0.131, 0.023)

### 3.4. Interaction of Hypertension and IR with Serum FFA Levels

To analyze the interaction between hypertension and IR on serum FFA levels by analysis of covariance. Conversely, this interaction did not exist by using analysis of covariance (SBP, DBP and IR as covariates, FFA as dependent variable), indicating that the relationship between FFA levels and hypertension or IR is independent, with no interference between hypertension and IR (Table 4).

**Table 4:** Interaction analysis by analysis of covariance.

Source	Type β Sum of Squares	df	Mean Square	*F*	Sig.
Corrected Model	1.074 ^a^	5	0.215	3.348	0.006
Intercept	0.736	1	0.736	11.462	0.001
SBP	0.022	1	0.022	3.745	0.048
DBP	0.377	1	0.377	5.876	0.016
IR	0.256	1	0.256	3.986	0.046
IR * SBP	0.012	1	0.012	1.220	0.270
IR * DBP	0.143	1	0.143	1.189	0.277
Error	28.751	448	0.064		
Total	168.672	454			
Corrected Total	29.826	453			

Note: ^a^ R squared = 0.036 (Adjusted R squared = 0.025).

### 3.5. Multivariate Logistic Regression Analysis

To exclude the influence of confounding factors (age, gender, BMI, TGs, TC, HDL-C and LDL-C) on the relationships among FFA, hypertension and IR, we used multivariate logistic regression analysis with hypertension and IR as the dependent variables and age, gender, FFAs, BMI, TGs, TC, HDL-C and LDL-C as the independent variables. FFA remained associated with hypertension and IR after controlling for age, gender, FFAs, BMI, TGs, TC, HDL-C and LDL-C (Table 5).

**Table 5:** Multivariate logistic regression analysis of FFA levels with hypertension and IR.

Variable	Index		β	SE	Waldχ^2^	*p*	OR	95%CI for OR
Hypertension	FFA (mmol/L)	<0.40	-	-	-	-	-	-
		0.40～	-0.41	0.61	0.446	0.504	0.66	(0.20, 2.21)
		0.62～	0.96	0.59	2.599	0.107	2.60	(0.81, 8.30)
		0.79～	1.878	0.65	8.273	0.004	6.54	(1.82, 23.52)
HOMA-IR	FFA (mmol/L)	<0.40	-	-	-	-	-	-
		0.40～	1.54	0.62	6.111	0.013	4.67	(1.38, 15.84)
		0.62～	1.55	0.59	6.801	0009	4.72	(1.47, 15.16)
		0.79～	1.69	0.58	8.250	0.004	5.40	(1.71, 17.06)

## 4. Discussion

Uyghurs represent one of the main ethnic groups in Xinjiang. Their unique geographical environment and living habits differ greatly from the rest of the country. In particular, their diet is quite distinct; the staple food is Nang, and their diet is rich in salt, with reduced fruit and vegetable intake. Moreover, their economic conditions are poor, the environment is harsh, their education and medical knowledge is deficient, and their self-awareness regarding disease prevention and treatment is severely lacking. Our previous studies showed that FFA levels, IR, and hypertension prevalence differed from those of other local ethnic groups [8,9,10,11]. Many domestic and foreign studies have confirmed that high FFA levels are related to the incidence of hypertension and IR [4,12,13].

In our study, we found that FFA levels were higher in the IR only group than in the normal group, suggesting that a high FFA level is associated with IR among Uyghurs. FFAs can affect insulin secretion by inhibiting glucose oxidation, leading to cell dysfunction, altered gene expression, and eventually IR. Some studies have shown that lower FFA levels can improve insulin sensitivity, and many drugs such as acipimox, hydrochloric acid, and thiazolines (TZDs) can improve IR by reducing FFA levels [14].

De Jongh *et al.* found that FFA levels modulate microvascular function and may contribute to obesity-associated IR, hypertension, and microangiopathy [15]. In our study, the subjects with hypertension exhibited only higher FFA levels compared to normal individuals, suggesting that a high FFA level may be related to the incidence of hypertension. Additionally, the FFA levels in the group with hypertension and IR were higher than in the normal group. Moreover, to eliminate the interference of age, BMI, TGs, TC, HDL-C and LDL-C, we compared FFA levels among the four groups using analysis of covariance, and differences were still found. The relationships of FFA with hypertension and IR also remained after controlling for age, gender, BMI, TGs, TC, HDL-C and LDL-C.

Additionally, as IR is the pathophysiological basis of MS and its associated conditions, including hypertension, obesity and dyslipidemia [16], IR is related to hypertension and FFA. Nonetheless, it remains unclear whether the relationship between hypertension and FFA is independent or dependent. Guo SX *et al.* found that high FFA levels were an independent risk factor for hypertension among Kazakhs [11]. In our study, because the effect of hypertension and IR did not equal the sum of the individual effects of hypertension and IR, we analyzed the interaction between hypertension and IR on serum FFAs by analysis of covariance and found that the interaction did not exist after balancing for other factors. Thus, we conclude that high FFA levels are also an independent risk factor of hypertension and IR among Uyghurs.

## 5. Conclusions

Among the Uyghur population, high FFA levels are an independent risk factor for the occurrence of hypertension and IR. Even in patients with hypertension who simultaneously have IR, FFA levels will be significantly increased. To reduce the prevalences of hypertension and IR, improving the behavioral lifestyle of this group through education or using certain pharmaceuticals to control FFA levels in the hope of reducing blood pressure and IR to ultimately prevent the incidence of MS and other related diseases should be considered.
